# Photoacoustic detection of propofol in breath gas for monitoring depth of anaesthesia: from bench to bedside

**DOI:** 10.1016/j.bja.2025.07.080

**Published:** 2025-09-09

**Authors:** Agnes S. Meidert, Péter Rucz, Judit Angster, Joseph Esser, András Miklós, Gustav Schelling

**Affiliations:** 1 Department of Anaesthesiology, University Hospital LMU Munich, Munich, Germany; 2 Fraunhofer Institute for Building Physics, Department of Acoustics, Group of Musical and Photoacoustics, Stuttgart, Germany

**Keywords:** breath gas analysis, depth of anaesthesia, monitoring, patient safety, propofol, volatile anaesthetic

## Abstract

**Background:**

Ensuring adequate depth of i.v. anaesthesia by measuring propofol in breath gas could increase patient safety. Mass spectrometry, representing the reference standard of propofol breath gas measurements, is not feasible in routine clinical practice; hence, a photoacoustic sensor was developed.

**Methods:**

The photoacoustic sensor quantifies propofol concentration in gas via the sound waves emitted by propofol molecules excited by light of specific wavelength and frequency. We studied the performance of the new sensor in propofol test gas, gas sampling bags filled with breath gas from different patients, and performed real-time measurements in patients undergoing propofol anaesthesia in comparison to ion-molecule reaction mass spectrometry.

**Results:**

In test gas, photoacoustic and mass spectrometry correlated with an *R*^2^ of 0.9975 in a range from 2.5 to 60 ppb. In gas sampling bags, propofol could be detected with both methods. Bland–Altman analysis of propofol general anaesthesia over 18 h in 10 patients revealed a mean propofol difference of −0.02 ppb (standard deviation 3.31) between mass spectrometry and photoacoustic measurements in breath gas, ranging from 4 to 47 ppb.

**Conclusions:**

Photoacoustic measurement of propofol concentration in breath gas is feasible with high accuracy in clinical applications.

**Editor’s key points**

• This combined laboratory and clinical study evaluated a novel photoacoustic method for monitoring propofol concentrations in exhaled gas, an approach to monitoring depth of anaesthesia.
• Photoacoustic measurements of propofol concentration in exhaled gas were feasible and accurate in clinical applications compared with standard measurements by mass spectrometry.
• Propofol concentration can be accurately measured by the photoacoustic method in breath gas of healthy patients in a clinical setting. Further optimisation and validation in patients with comorbidities might validate the method as a routine monitor for depth of propofol general anaesthesia.

Maintenance of general anaesthesia frequently uses volatile anaesthetics or i.v. anaesthetics, usually propofol. The concentrations of volatile anaesthetics are continuously monitored in inhaled and exhaled breath by gas analysers. This technology is embedded in most anaesthesia machines to assist in monitoring depth of anaesthesia.^1^ For propofol, there is currently no equivalent measurement device, which remains an unsolved problem in modern anaesthesia.

A small fraction of injected propofol is eliminated via the lungs and can be detected in exhaled gas.^2^ Propofol in exhaled gas closely reflects its anaesthetic effect on the brain,^3^ usually determined by mass spectrometry (MS) in this context.^4^^,^^5^ However, such apparatus is not feasible for the use in an operating room because of its sensitivity, size, technical requirements, and cost.

We tested whether a small, relatively inexpensive detector based on the photoacoustic principle could measure propofol in breath gas during anaesthesia. Photoacoustic detection of molecules in gas samples is used for monitoring industrial fumes by quantifying a specific substance by laser light impulses that lead to an acoustic signal. With light-emitting diode (LED) light sources instead of lasers and small microphones developed for smartphones, we designed a photoacoustic sensor prototype to detect propofol in breath gas. This novel technology has the potential to become a clinically feasible solution for continuous monitoring of propofol in breath gas. Here, we evaluated the accuracy of photoacoustic measurements of propofol in test gas and patients' breath during propofol anaesthesia, compared with ion-molecule reaction MS as reference standard.

## Methods

The study had three parts: 1) laboratory experiments with propofol test gas, 2) off-line patient breath gas analysis, and 3) online analysis of patient breath gas during propofol anaesthesia in the operating room. For all experiments, the reference method was ion-molecule reaction MS, and the test method was the photoacoustic sensor.

### Development of a photoacoustic sensor for propofol gas detection

**Fig 1:**
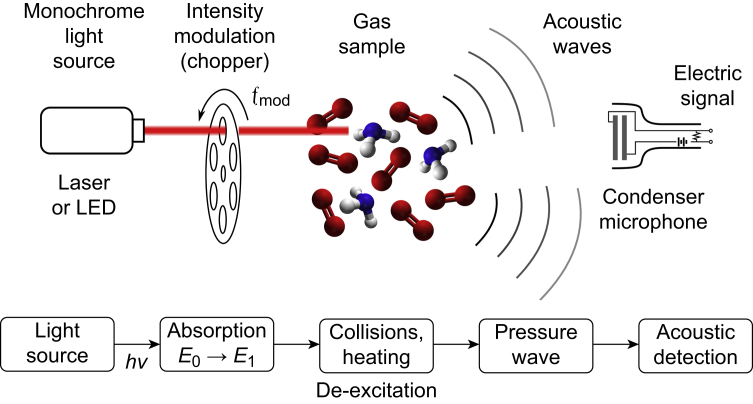
Principles of photoacoustic detection. LED, light-emitting diode.

The technique of photoacoustic spectroscopy is based on absorption of light of defined wavelength by a specific molecule in a gas sample. By modulating the intensity of the light at a given frequency, the absorption and de-excitation causes periodic local heating and cooling of the gas sample, which results in pressure waves. These waves can be recorded as sound waves by sensitive microphones, resulting in an electric signal. The greater the absorption and sound, the higher the concentration of the molecule. Figure 1 illustrates the technical principle of photoacoustic detection. As a light source, high-energy LEDs can be used instead of lasers. The intensity modulation can be implemented electronically rather than mechanically. Propofol exhibits a strong peak in its light absorption spectrum near 275 nm,^6^ therefore our photoacoustic sensor irradiates the gas sample at this wavelength.

**Fig 2:**
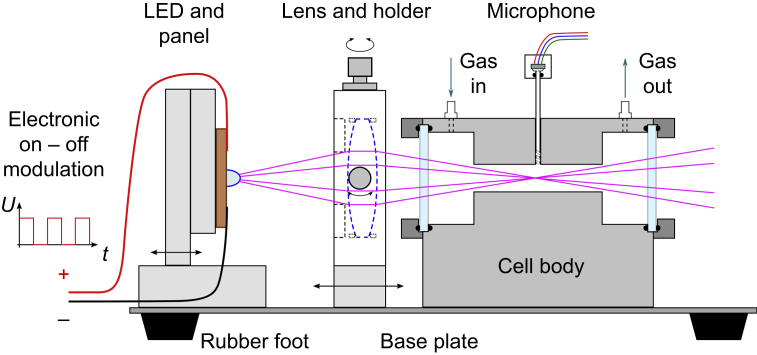
Photoacoustic sensor setup. Light with electronically modulated intensity from LEDs passes through the cell. LED, light-emitting diode.

Patients undergoing anaesthesia with i.v. propofol exhale very small concentrations of propofol (∼10 ppb).^3^^,^^7^ Therefore, monitoring propofol concentration in the breath must be very sensitive (the limit of detection should be at least 1 ppb). Propofol readily adsorbs onto surfaces. To solve this problem, surfaces are made from covered steel, and constantly heated to >100°C. Furthermore, the light beam of the LED is prevented from hitting the walls of the measurement cell by optical lenses, thereby only exciting molecules in the gas phase (Fig. 2).

The form and size of the cell was chosen to optimise the acoustic properties with two buffers at each end.

The modulation frequency at which the photoacoustic signal is maximal, and thus the sensitivity is optimal, strongly depends on the temperature and composition of the gas through its effect on the speed of sound. In breath gas measurements in patients having surgery, oxygen concentration is the most important factor. As the photoacoustic cell can amplify acoustic waves by their constructive interference, the optimal detection frequency coincides with the acoustical resonance frequency of the cell. We developed dedicated hardware and software elements to continuously follow the changes in resonance frequency resulting from variations in gas composition to automatically adjust the modulation frequency. The frequency tracker has a single calibration constant, which is determined under laboratory conditions. *In situ* measurements are performed without adjusting this calibration value or knowing the actual oxygen concentration at any time during measurement.

### Differentiation of propofol and acetone

Propofol has its absorption peak in the UV range near 275 nm.^6^ However, there is a strong light absorption overlap with acetone in that range, therefore the sensor has a second chamber in which the gas sample is excited at 295 nm, which is specific for acetone. By taking the difference of the two signals, propofol concentration can be evaluated. For a typical concentration of propofol in breath of 10 ppb and acetone of 0–10 ppm, and taking into account our actual sensitivities of ∼25 μV ppb^−1^ for propofol and 160 μV ppm^−1^ for acetone, the magnitudes of the electric signal components resulting from propofol and acetone are on a similar scale.

### Laboratory experiments with test gas

**Fig 3:**
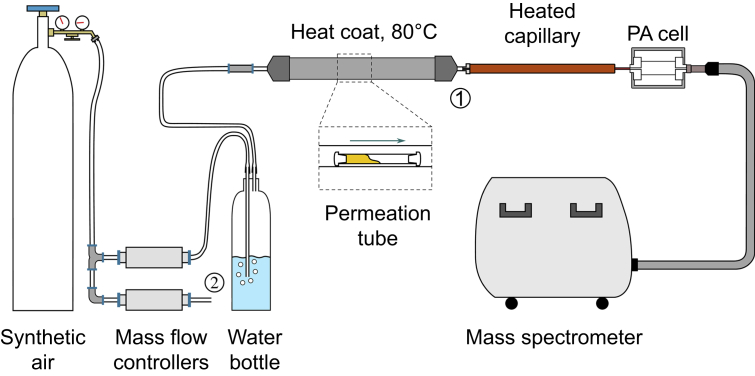
Experimental setup of simultaneous photoacoustic and mass spectrometric propofol test gas measurements. Sampling points (1) and (2) allow measurement of propofol gas and pure synthetic air, respectively, at controlled flow rates. PA, photoacoustic.

The reference detection method for propofol in exhaled breath is MS. We used an MS system based on ion-molecule reaction (Airsense, V&F, Absam, Austria), as described.^4^ Calibrations of the MS were performed with propofol test gas (0.8 μl liquid pure propofol in 1500 L nitrogen and 10 L argon [propofol 68 ppb], Messer, Gumpoldskirchen, Austria). Acetone calibration of the MS was performed by the manufacturer with acetone test gas (5000 ppb) and subsequently calibrated daily via indirect calibration via Isopren test gas. In order to measure different concentrations and the ability to track concentration changes of propofol, two permeation tubes with differently sized surfaces were constructed (Fig. 3). By adjusting the flow rate of synthetic air through the tube, different concentrations were obtained. After calibration measurements with MS, different propofol concentrations were measured with the photoacoustic sensor and MS simultaneously. Flow rates were set at 400, 200, and 100 ml min^−1^ to achieve concentrations ranging from 5 to 60 ppb for 15 min at each concentration. The experiment was repeated six times. Response time (T90) was calculated as the time needed to reach 90% of a stable plateau after connecting the capillary to sampling point (1), and after connecting back to synthetic air at sampling point (2) to reach 10% of the plateau value. Repeating the experiment multiple times also allowed for assessing hysteresis.

### Measurements in patients

All patients included in breath gas analyses gave informed consent. The local ethics committee (Ethikkommission bei der Medizinischen Fakultät der LMU München) approved the study (protocol 20-696).

### Off-line analysis of breath gas samples from patients having propofol anaesthesia

Tedlar™ bags made from polytetrafluoroethylene (PTFE) were used. Conventional Tedlar™ bags made from polypropylene produced a photoacoustic signal coming from the material. In addition, high adsorption of propofol breath gas on polypropylene prohibited valid measurements.

Breath gas of three intubated and mechanically ventilated patients having i.v. anaesthesia with propofol for scheduled surgery was sampled in PTFE Tedlar™ bags. First, an inspiratory hold was achieved by occluding the breathing circuit after inspiration. The first 50 ml of expiratory gas were discarded to minimise capturing expiratory gas from the anatomical dead space. By opening the valve to the Tedlar™ bag, the remaining air in the lungs flowed passively into the bag. This procedure was repeated until the bag was filled with 3 L of exhaled gas. Inspiratory oxygen was set to 100%. Vital signs were closely monitored during breath gas sampling. In one patient, the volatile anaesthetic sevoflurane was present in addition to propofol. In another patient, a second sample was taken at inspiratory oxygen 40% to evaluate changes in frequency.

The expiratory gas samples were analysed in the laboratory shortly after sampling to minimise diffusion through walls and adsorption. Measurements had to be taken consecutively with MS and photoacoustic sensor because only one device could be connected to the sampling bag. After photoacoustic measurements, a second measurement by MS was performed to assess the stability of the measured concentrations. Photoacoustic signals were transformed from μV to ppb (propofol) and ppm (acetone) concentrations based on the sensitivities evaluated from the test gas measurements for online monitoring in the operating room.

### Online monitoring of breath gas in patients having propofol anaesthesia

Measurements were performed in the operating room of the LMU University Hospital in 10 female patients undergoing surgery using propofol as anaesthetic. Blood pressure, ECG, and pulse oximetry were monitored. After induction with sufentanil, propofol, and rocuronium, tracheal intubation was performed. The photoacoustic sensor and the MS were connected to the distal end of the tracheal tube via an X-piece. The photoacoustic sensor and MS analysed mixed inspiratory and expiratory gas with a continuous side-stream flow rate of 100 ml min^−1^ and 63 ml min^−1^, respectively. The calculated time for the breath sample to pass through the cell was 30 s. Pressure control ventilation was adjusted to achieve an end-tidal CO_2_ between 4.7 and 5.3 kPa, and positive end-expiratory pressure was set to 5 mbar. For induction and emergence of anaesthesia, inspiratory concentration of oxygen was 100% and fresh gas flow was 8 L min^−1^, which was reduced to inspired oxygen concentration of 40% and 4 L min^−1^ fresh gas flow for maintenance of anaesthesia. Processed electroencephalogram (EEG) recording patient state index (PSI, with a target range for anaesthesia of 25–50) and suppression rate was used in all patients (SedLine®, Masimo, Irvine, CA, USA). All patients received a continuous infusion of propofol via syringe pump. Propofol dosage of induction, maintenance and intermittent boluses when clinically indicated (e.g. patient movement or increase in PSI) were at the discretion of the attending anaesthesiologist.

### Data processing and statistical analysis

Matlab R2018b (The MathWorks Inc., Natick, MA, USA) and R (2024 The R Foundation for Statistical Computing, Vienna, Austria, V 4.4.2) were used for data processing, statistical analysis, and graphical presentation. Data from MS were collected via dedicated software (V&F Server and Client, V&F). Photoacoustic data were recorded using software developed in-house which used lock-in processing of microphone signals. It was validated and tested during laboratory measurements. Processing results were validated by comparison to an SR-850 lock-in amplifier (Stanford Research Systems, Sunnyvale, CA, USA).

Anaesthesia data including vital signs, dose of propofol, EEG data, and ventilator settings were processed via the electronic anaesthesia protocol (Narkodata, Imeso, Gießen, Germany). Continuous data describing patient characteristics are presented as median (interquartile range), and intraoperative data as mean (standard deviation [sd]).

For test gas measurements via permeation tube and sampled patient breath gas, photoacoustic sensor (μV) and MS (ppb) signals were analysed via least squares linear regression. For online patient data of propofol and acetone, values were averaged over 10 s for analysis. For EEG data, one value every 10 s was analysed.

Both methods were compared via Bland–Altman analysis accounting for repeated measurements.^8^ Mean bias, sd, and 95% limits of agreement are presented in ppb (propofol) and ppm (acetone). For tracking changes in propofol breath gas concentration and the corresponding processed EEG, 100 s before and 400 s after bolus injections of propofol were statistically analysed with the time point of bolus injection as reference concentration.

## Results

### Bench experiments with test gas

**Fig 4:**
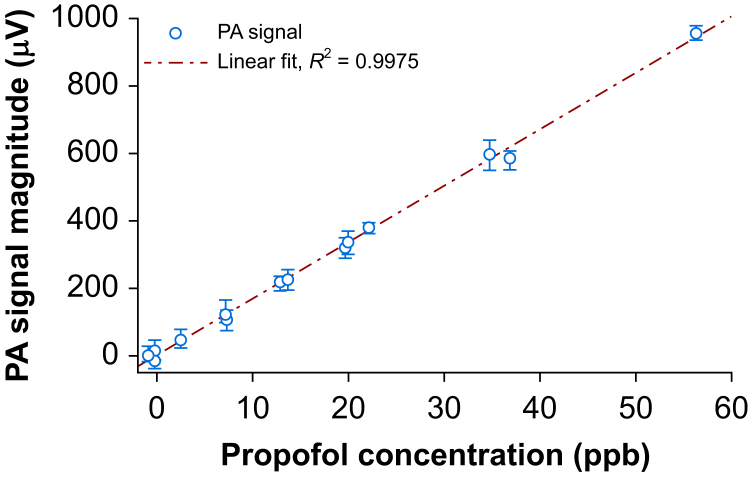
Propofol test gas from permeation tube measured with mass spectrometry in ppb and photoacoustic sensor in μV. Whiskers denote minimal and maximal values. PA, photoacoustic.

We used permeation tubes to test the ability of the photoacoustic sensor to obtain a propofol signal in different ranges of concentrations compared with MS. At different flow rates, concentrations of propofol ranging from 2.5 to 60 ppb were achieved. The photoacoustic signal in μV and the MS signal in ppb correlated linearly with an *R*^2^ of 0.9975 (Fig. 4). These measurements were used to translate photoacoustic signals in ppb concentrations. By repeatedly switching between pure synthetic air and a fixed propofol concentration provided by the permeation tube system, we found the T90 rise time as 1.5 min and the fall time as 1.2 min with a flow rate of 200 L min^−1^. In the same experiment, we also assessed hysteresis effects and found no changes in the steady-state levels when repeating the experiment. After completion of a series of propofol measurements, we heated the sensor to an elevated temperature and flushed it with synthetic airflow while measuring the gas at the outlet of the cell by MS. We found no adsorbed propofol retained by the photoacoustic cell, even after several days of measurements.

### Off-line analysis of exhaled gas

Exhaled gas from anaesthetised patients was collected in suitable bags. Four samples of breath gas from three patients shortly after induction of anaesthesia were analysed. Propofol could be measured successfully by MS and the PA sensor in three samples. Samples 1, 2, and 3 had MS propofol concentrations of 10, 10, and 9 ppb and PA propofol signals of 720, 698, and 612 μV, respectively. The presence of the volatile anaesthetic sevoflurane in another sample caused a detuning of the acoustic resonance preventing meaningful photoacoustic measurements because of the high molecular weight of sevoflurane. Because of adjustments in acetone sensitivity, μV values for propofol of the photoacoustic sensor did not match test gas measurements.

### Online monitoring during surgery

Data collected from 10 mechanically ventilated patients undergoing propofol anaesthesia were analysed (see Table 1 for patient characteristics). Episodes of technical problems such as incomplete connection of devices to the tracheal tube because of positioning of the patient were excluded. Frequency tracking was used to adjust for the influence of different inspired oxygen concentrations on the speed of sound.

**Table 1:** Patient characteristics and propofol administration, shown as median (interquartile range) or mean (standard deviation).

	
Age (yr)	49 (41.5–65.5)
Height (cm)	169.5 (165.25–170)
Weight (kg)	62.5 (59.25–65)
Duration of anaesthesia (min)	157 (72)
Continuous propofol dose (mg)	940.64 (553.63)
Induction propofol dose (mg)	173.00 (33.35)
Propofol adjustment dose (mg)	99.00 (63.50)
Continuous propofol dose (mg min^−1^)	6.61 (2.66)
Weight adjusted continuous propofol dose (mg min^−1^ kg^−1^)	0.10 (0.05)
Weight adjusted total propofol dose (mg min^−1^ kg^−1^)	0.13 (0.06)

**Fig 5:**
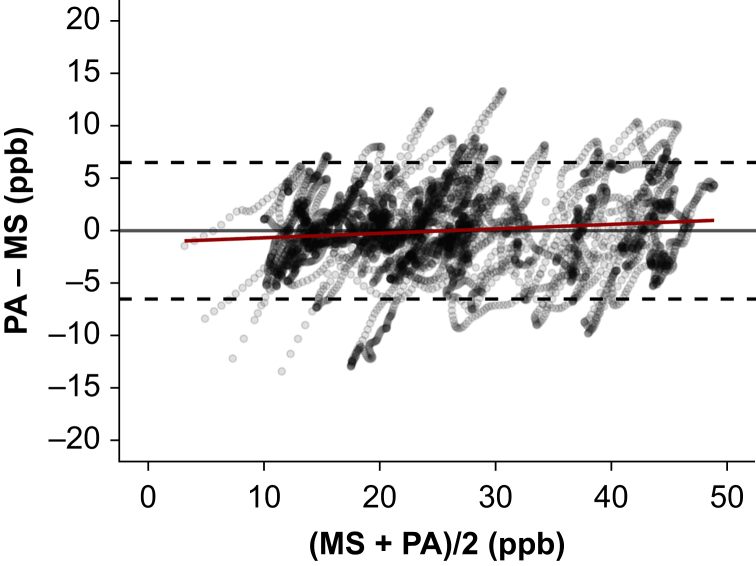
Evaluation of accuracy and precision for propofol. Bland–Altman diagram of propofol concentrations in mixed breath gas measured by the reference method mass spectrometry (MS) and the photoacoustic test method (PA) in ppb. Mean bias, solid line; upper/lower 95% limits of agreement, dashed lines; linear regression, red line.

Averaging over 10 s, 6557 total data points of breath gas propofol measurements were available for analysis. This translates to 18.2 h of anaesthesia. The Bland–Altman analysis revealed a mean difference (sd) between mass spectrometer and photoacoustic sensor of −0.02 ppb (3.31) (Fig. 5). The 95% limits of agreement were −6.51 to 6.47 ppb with measurements ranging between 3.9 and 47 ppb. Bland–Altman analysis of 6070 acetone measurements showed a mean difference of −0.013 ppm (0.57, −1.12 to 1.10, Supplementary Fig. S1).

**Fig 6:**
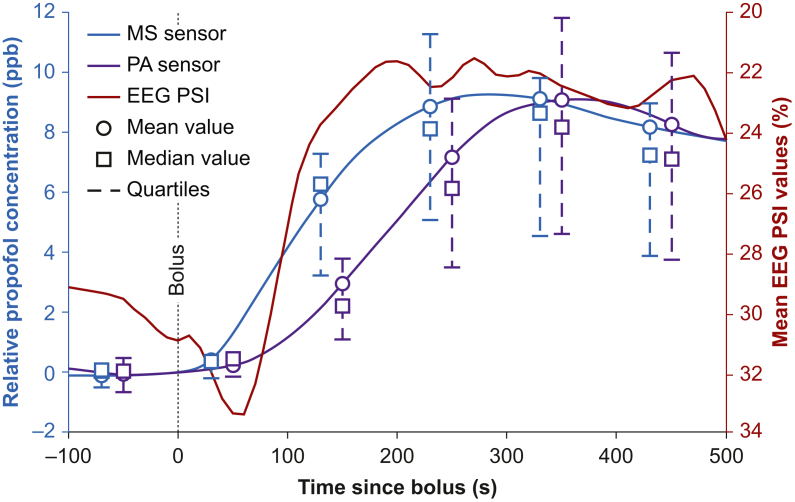
Adjustment of propofol dose because of insufficient depth of anaesthesia; average of 13 episodes in eight patients. Graphical evaluation of changes in breath gas propofol concentration after administration of an i.v. propofol bolus determined with mass spectrometry (MS, blue) and photoacoustic sensor (PA, purple) in relation to changes in processed EEG (red). Circles are mean values, boxes are median, and whiskers interquartile range. PSI, patient state index.

During anaesthesia, in eight patients 13 clinically indicated adjustments of the administered propofol dosages were necessary because of propofol underdosing. Patient movement indicating insufficient depth of anaesthesia occurred frequently despite processed EEG suggesting adequate sedation. Figure 6 displays the subsequent change of propofol concentration and corresponding changes in EEG. Initial propofol concentrations were normalised to 0 to show relative changes of propofol concentration in breath gas after dosage adjustment when a bolus of i.v. propofol was applied. For propofol measurements, mean, median, and quartiles are presented. For PSI, the high variance prevented a meaningful graphical presentation of quartiles, therefore only mean values are shown in Figure 6.

**Fig 7:**
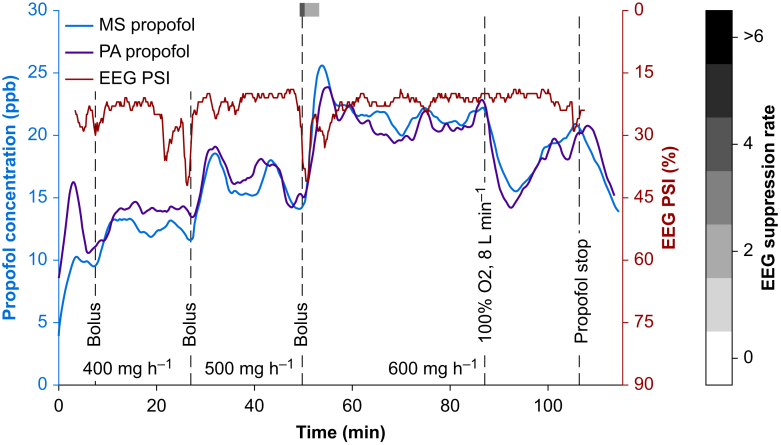
Time course of one exemplary patient during surgery. Breath gas propofol concentrations measured by mass spectrometer (MS, blue) and photoacoustic sensor (PA, purple) with corresponding relative changes in processed EEG patient state index (PSI, red) and suppression rate in % (grey) on top. Bolus indicates an additional dose before adjustment of continuous infusion rate.

Figure 7 shows the measurements of one exemplary patient during surgery with propofol in breath gas, PSI, and suppression rate from processed EEG. Three adjustments of propofol dosage were necessary in this patient, reflected by an increase of propofol concentration in the breath gas. Doubling the flow rate of fresh gas at the anaesthesia machine from 4 L min^−1^ to 8 L min^−1^ resulted in a transient dilution of measured propofol concentration (minute 86).

## Discussion

We describe measurement of propofol in test gas and exhaled patient breath during general anaesthesia using a photoacoustic technique. Compared with the reference standard ion-molecule reaction MS, the photoacoustic sensor can detect propofol in the low ppb range with high accuracy and precision. In addition, changes in propofol concentration in breath gas are reliably detected by photoacoustic measurements.

Monitoring effective propofol concentrations during anaesthesia is highly clinically relevant. Various groups have worked on propofol breath gas monitoring in anaesthetised patients since 2003.^9^ Hornuss and colleagues^3^ demonstrated that breath concentration reflects the effect of propofol on processed EEG and correlates with propofol plasma concentrations.^10^ In addition, a recent study in mice showed a strong correlation between propofol concentrations in breath and brain.^11^ Therefore, it seems plausible to interpret steady-state propofol breath gas concentrations as a noninvasive monitor of anaesthesia depth, analogous to minimum alveolar concentration (MAC) for volatile anaesthetics. We were able to confirm this general finding: there was a close alignment between changes in propofol breath gas and changes in processed EEG. Propofol pharmacokinetics and pharmacodynamics in individual patients vary considerably, precluding the use of a fixed dosing regimen. In our study, changes in propofol concentrations reflecting additional propofol dosages in case of involuntary patient movements (a far more frequent occurrence during anaesthesia than awareness) were reliably detected.

In clinical research, MS is the reference method for propofol quantification in breath gas. This technique is expensive, and the machines are large, noisy, and require frequent calibration. Therefore, real-time monitoring of propofol in breath gas using MS is not feasible for routine clinical use.^5^ Ion mobility spectrometry (IMS) promises to be a more practical solution. A bedside monitor based on IMS was developed, however humidity in human breath affected measurement performance.^12^ Laurila and colleagues^6^ first described the detection of propofol under laboratory conditions by photoacoustic spectroscopy using a high-power laser. However, lasers are expensive and sensitive, which limits their utility for routine clinical use. To overcome this problem, we used a novel photoacoustic approach. During development, several measures were taken to ensure practicability. The photoacoustic sensor is small enough to fit into the anaesthesia workplace (size of a shoebox). All components are low cost: laser technology has been replaced by LEDs as light source, and miniaturised microphones developed for mobile phones are used. In addition, no test gas calibration is necessary in daily routine. Therefore, the prototype meets many of the requirements to become part of routine monitoring.

Another technical obstacle is propofol adsorption to surfaces. To avoid adsorption problems within the photoacoustic sensor, all connecting tubes and inner surfaces are heated. In addition, the light beam only excites molecules in the gas phase. The side-stream sampling is performed at the patient’s side of the breathing circuit filter that adsorbs most of the exhaled propofol. We cannot rule out a certain degree of adsorption of propofol on the tracheal tube made from polyvinyl chloride. However, the inner surface of the tracheal tube is relatively small in relation to the high volume of breath (high number of molecules) passing through, and the expected early saturation of its surface with propofol presumably makes the effect negligible.

Our sensor uses a continuous low-flow side-stream for sampling, as do most techniques described above. In the case of the photoacoustic sensor, it takes ∼30 s for the gas sample to fill the full internal volume of the cell of 50 ml, which only enables analysing mixed breath gas. As the vacuum chamber of the MS is much smaller, separate sampling of inspired and expired samples would be possible by MS. Nevertheless, the small but not negligible transient caused by adsorption–desorption to the walls of the sampling capillary and the molecule count cycles not being time-synchronised with respiration prevents accurate differentiation between inspiratory and expiratory propofol (and acetone) concentrations. Therefore, we analysed mixed breath gas with both methods. It can be argued that alveolar gas samples would be preferable to mixed breath gas. However, the concentrations measured in our patients by both methods in mixed gas samples were higher than previously reported.^3^^,^^7^ We suppose that this result stems from sampling the breath gas by connecting the low-adsorption capillaries as close to the patient as possible in our setup. It requires further studies with a standardised setup controlled for adsorption to establish reference values for exhaled propofol, and how to correct for mixed gas sampling.

Ensuring adequate response time is of key importance in the clinical application of the sensor. In laboratory experiments with well-defined flow rates and concentrations, we found response times adequate for measuring patients’ breath gas. The response time of the photoacoustic sensor mainly depends on the sampling flow rate: by increasing the flow, the response time decreases and *vice versa*.

Besides propofol, other components are part of exhaled breath, such as varying fractions of oxygen and volatile anaesthetics. As the chemical properties of the gas species influence the speed of sound, varying the proportions of these components causes shifts in the acoustic resonance frequency of the photoacoustic cell. Presence of the volatile anaesthetic sevoflurane made meaningful photoacoustic measurements in bags with breath gas samples impossible, as sevoflurane is a heavy molecule. Therefore, for online measurements in patients, a frequency tracker was used. It reacts automatically to the change in speed of sound, mainly caused by varying oxygen content, in breath gas. For clinical use, inability to measure propofol concentrations in the presence of sevoflurane would be a major limitation of any photoacoustic sensor. Future studies should validate an extended range of frequencies to detect the shifts and adjust for the presence of sevoflurane in exhaled gas. Other components of breath gas come from the patient, mainly acetone but also other volatile organic compounds. Laurila and colleagues^6^ showed that there is considerable overlap of light absorption in the mid-infrared spectral region for water, acetone, isoprene, and CO_2_. In the ultraviolet region, there is only overlap with acetone, but its light absorption is >100 times weaker than that of propofol. This is important because >1000 ppb acetone can be present in breath. In typical breath gas composition, acetone produces nearly the same signal magnitude in our photoacoustic sensor as propofol. Thus, by using two acoustically identical resonant chambers with different wavelengths of UV light, we can correct for different acetone concentrations in exhaled gas.

There are many open research questions before propofol breath gas monitoring can be translated to clinical routine. One is the lack of reference values for adequate anaesthesia. A larger study with simultaneous evaluation of propofol plasma concentrations in different patient groups is needed to establish reference values for breath gas-guided anaesthesia. It is also possible that because of the individual differences in brain susceptibility, there are no absolute cut-off values, and that determining the trend of propofol in breath gas over the course of anaesthesia is more important than absolute numeric values. The relatively broad range of propofol values in our study supports this assumption. We were able to detect an increase of propofol in breath after an increase in propofol dose and a decrease in breath gas concentrations after stopping propofol infusion. Another question is the influence of lung disease or pulmonary shunting on propofol in breath gas: immobilised patients are prone to developing atelectasis, leading to blood passing through the lungs without participating in gas exchange. Finally, there are no data on the influence of conditions causing altered perfusion of the lungs, such as heart disease or pulmonary hypertension, on propofol concentrations in breath gas.

Our study has some limitations. Our sensor is a prototype for initial clinical evaluation of the measurement principle. Before the photoacoustic technique can be transferred to the anaesthesia workplace, the mechanical stability of the sensor must be improved. During clinical measurements, insufficiently tight connections represented a problem. The laboratory data were collected under controlled circumstances without the presence of acetone or electrical noise from devices in the operating room. A potential source of measurement bias is the use of a single MS as reference technique for all measurements. Although calibration was performed carefully, relying on a single machine can influence generalisability. We did not systematically assess the impact of different settings of the anaesthesia machine (e.g. flow rate). Furthermore, we studied 10 healthy female patients for the clinical evaluation. Extrapolations of these results to patients with obesity, severe pre-existing illnesses, or frail and old patients is therefore impossible and must be the subject of further studies.

Future studies are needed to compare photoacoustic values to plasma concentrations for further evaluation of accuracy. Studying diverse patient populations and clinical situations is key to a broad application of the technique in clinical routine. In addition, further development of the prototype to meet all requirements for regulatory approval is another step toward clinical translation.

Taken together, our findings demonstrate that propofol can be reliably measured by the photoacoustic method in breath gas of patients in a clinical setting, and has the potential to become a routine monitor for depth of propofol general anaesthesia.

## Authors’ contributions

Conceptualisation: ASM, PR, JA, AM, GS

Methodology: ASM, PR, AM, JA, GS

Investigation: ASM, PR, AM, JE

Visualisation: ASM, PR

Funding acquisition: GS, JA, ASM, AM

Project administration: GS, JA

Supervision: GS, AM, JA

Writing of original draft: ASM, PR

Manuscript review and editing: ASM, PR, GS, JE, JA, AM

## Data and materials availability

All data are available from the authors upon request.

## Funding

Supported by Bundesministerium für Wirtschaft und Klimaschutz (KF2664503CS1); Bundesministerium für Bildung und Forschung (BMBF) (03VP07502); and Deutsche Gesellschaft für Anästhesie und Intensivmedizin, Stipendium Dierichs 2022.

## Declarations of interest

ASM, PR, JA, AM, and GS are co-inventors of a pending patent from their institutions with the title ‘Vorrichtung und Verfahren zur Erfassung von zumindest zwei Spurengasen in einer Ausatemluft’, European Patent Office 24174153.7. ASM has received speaker honoraria from Edwards Lifesciences (Irvine, CA, USA). JE declares that he has no conflict of interest.
