# Supplementary material for: Photoacoustic detection of propofol in breath gas for monitoring depth of anaesthesia: from bench to bedside
Source: Br J Anaesth. 2025 Sep 9;135(5):1203–11. doi: 10.1016/j.bja.2025.07.080 (PMC12597363; doi:10.1016/j.bja.2025.07.080)
Supplement: Multimedia component 1 [file mmc1.docx]

Figure S1


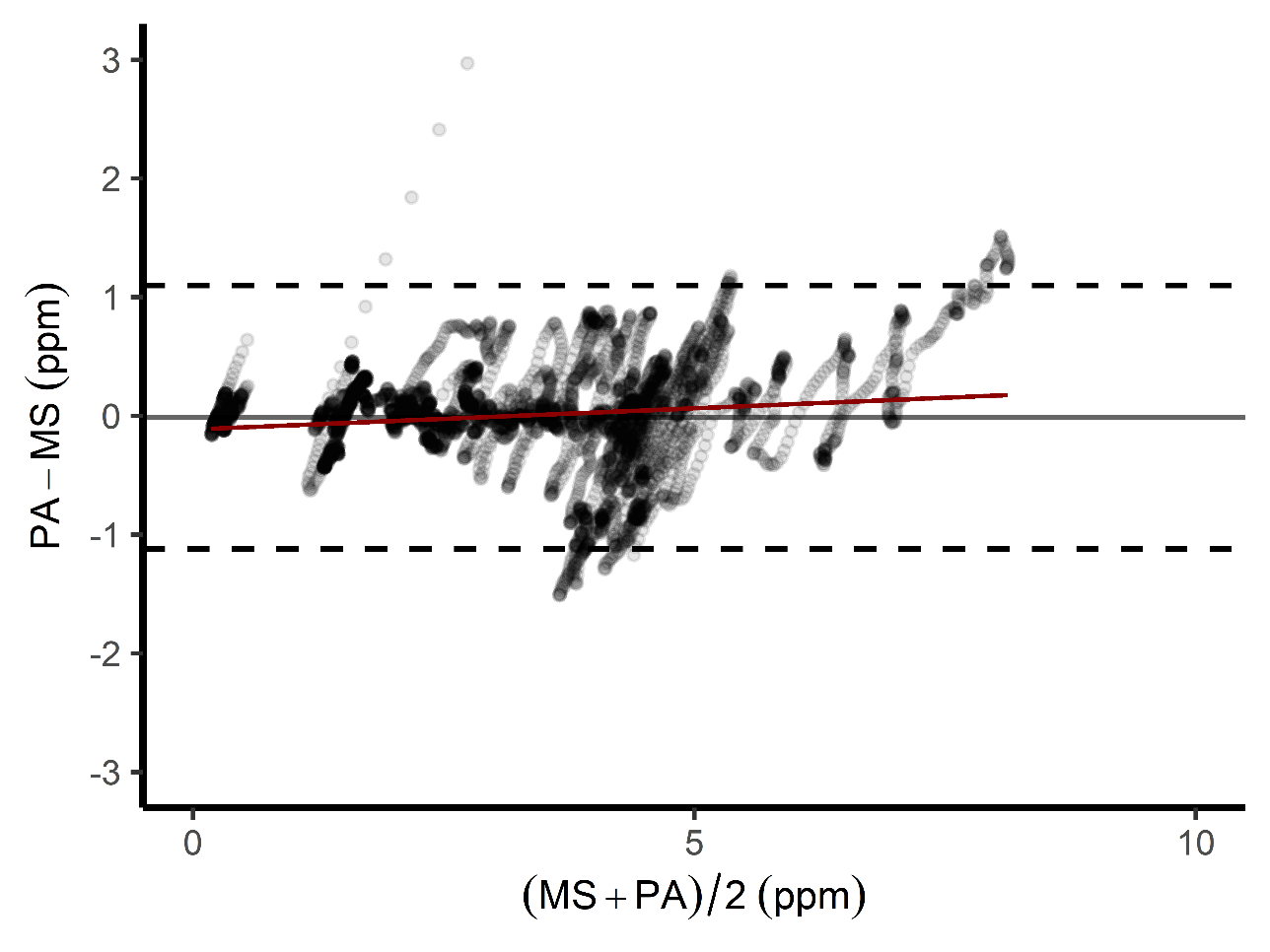


Figure 1 Evaluation of accuracy and precision for acetone: Bland-Altman diagram of expiratory acetone measured by the reference method mass spectrometry (MS) and the photoacoustic test method (PA) in ppm. Mean bias = solid line, upper/lower 95% limits of agreement = dashed lines, linear regression = red line
